# Artificial Intelligence-Assisted Structural Analysis of Bones with Paget’s Disease of Bone and Osteoporosis: Lessons from Mouse Models

**DOI:** 10.3390/diagnostics16162618

**Published:** 2026-08-18

**Authors:** Jie Liu, Shun-Yu Kan, Xiwen Xin, Tianle Chen, Henry Tseng, Yung-Chieh Hsu, Tai-Hsien Wu, Do-Gyoon Kim, Ching-Chang Ko

**Affiliations:** 1 Division of Orthodontics, The Ohio State University College of Dentistry, Columbus, OH 43210, USA; liu.7050@buckeyemail.osu.edu (J.L.); michaelkan55555@gmail.com (S.-Y.K.); xin.153@buckeyemail.osu.edu (X.X.); wu.5084@osu.edu (T.-H.W.); kim.2508@osu.edu (D.-G.K.); 2 Department of Orthodontics and Dentofacial Orthopedics, University of Colorado School of Dental Medicine, Aurora, CO 80045, USA; 3 Department of Computer Science, Boston University, 665 Commonwealth Avenue, Boston, MA 02215, USA; tianle@bu.edu; 4 Duke Eye Center, Duke University Medical Center, Durham, NC 27710, USA; henry.tseng@duke.edu; 5 Independent Researcher, 2580 S University Blvd #302, Denver, CO 80210, USA; kisdmars@gmail.com

**Keywords:** Paget’s disease of bone, osteoporosis, micro-computed tomography, artificial intelligence, random forest, convolutional neural network

## Abstract

**Background/Objectives:** Paget’s disease of bone (PDB) and osteoporosis are chronic metabolic bone disorders characterized by disrupted bone remodeling and increased skeletal fragility; however, the underlying mechanism of PDB remains poorly understood. Artificial intelligence (AI) has emerged as a transformative tool in medical imaging, enabling automated feature extraction and improved diagnostic classification of skeletal disorders. This study aimed to investigate whether AI could distinguish subtle variations in bone morphology between PDB and osteoporotic bone. **Methods:** C57BL/6 mice femurs were scanned by µCT: 16 optineurin-knockout mice with a PDB phenotype (20–26 months), 25 genetically matched wild-type Aging mice (20–26 months), and 15 ovariectomized (OVX) mice with osteoporotic bone phenotype (4.5 months). Two AI algorithms were investigated: a machine learning (ML) model using 22 µCT-derived features trained with a Random Forest (RF) classifier, and a deep learning (DL) model using a 3D convolutional neural network (3D-CNN) trained on raw µCT images. Leave-one-out cross-validation was applied to evaluate model robustness. **Results:** Significant differences in volumetric, density, and morphological parameters of cortical and trabecular bone were observed between PDB and osteoporosis (*p* < 0.05). The RF algorithm achieved 90% accuracy in distinguishing PDB from both aging- and OVX-induced osteoporosis and provided feature importance rankings that improved model interpretability. The 3D-CNN achieved classification accuracies of 70% for PDB vs. OVX and 68% for PDB vs. aging, demonstrating the feasibility of an image-based DL approach. **Conclusions:** AI-based RF and 3D-CNN models demonstrated promising performance in differentiating PDB from osteoporosis using µCT-derived bone features. These findings suggest potential for using AI to assist with analyzing skeletal images in the diagnosis of metabolic bone disorders.

## 1. Introduction

Paget’s disease of bone (PDB) and osteoporosis are two prevalent, age-dependent, metabolic bone disorders that disrupt bone remodeling and increase fracture risk; yet, their underlying disease mechanism remains unclear. Current efforts at studying these diseases and associated changes in bones have been hampered by a lack of a rapid, quantitative methodology for characterizing subtle bone changes at the morphological and structural levels. Because the human body has 206 bones and an average mouse has over 225 bones in the body, manual macroscopic and microscopic characterization of all the bones is prohibitively expensive and labor intensive [1,2,3,4]. We report here the development of an artificial intelligence (AI)-assisted methodology for rapid, automated characterization of bone changes directly from raw imaging results without the need for manual segmentation. This will help researchers rapidly characterize different bone phenotypes and can be translated into clinical applications to diagnose and assess therapeutic responses in PDB and osteoporosis patients.

PDB is age-dependent and affects 1- 2% of individuals over 55 years of age in the US [5,6], with a slight predominance in males [7]. It is characterized by localized regions of excessive bone turnover, initiated by hyperactive osteoclasts and followed by disorganized osteoblastic formation of abnormal new bone [8]. This aberrant bone remodeling results in structurally weaker bones with larger volume, disorganized architecture, and reduced mechanical strength [9,10]. Clinically, PDB can present with bone pain, deformities such as bowing of long bones, skull enlargement, and pathological fractures. Other non-skeletal complications include hearing loss due to auditory nerve compression by abnormal bone, nephrolithiasis, heart failure, and osteosarcoma [1,5,11]. There is no cure and treatment is limited to addressing specific symptoms.

The pathophysiology of PDB remains incompletely understood. Both environmental and genetic factors have been implicated [12], including paramyxoviruses infections [13,14], and mutations in genes such as Sequestosome 1 (SQSTM1), tumor necrosis factor receptor superfamily member 11a (TNFRSF11A), valosin-containing protein (VCP), and profilin 1 (PFN1) [2,15,16]. Among these, SQSTM1, which encodes the scaffold protein p62, is one of the most frequently identified genetic abnormalities in PDB and plays a critical role in regulating osteoclast signaling pathways, particularly NF-κB activation [15]. More recently, Optineurin (OPTN), has been genetically associated with PDB pathogenesis [13,17], through negative regulation of osteoclast differentiation [18,19]. Recent studies employing global Optn knockout (KO) mice have demonstrated a recapitulation of key clinical features of PDB [20], offering a promising preclinical platform for further investigation.

Osteoporosis is the most prevalent progressive metabolic bone disease, characterized by reduced bone mass and density. There is an imbalance between bone resorption and formation which clinically results in increased bone fragility and an elevated risk of fractures [21,22]. Osteoporosis is broadly classified into primary subtypes, resulting from postmenopausal and age-related changes [21,23]. Postmenopausal osteoporosis results from estrogen deficiency and predominantly affects trabecular bone in women [22]. In contrast, the senile/aging type of osteoporosis affects both men and women, and is characterized by reduced osteoblast activity, increased marrow adipose tissue, and diminished bone formation [22,23].

Bone loss is diagnosed by using standard X-ray radiographs, dual-energy X-ray absorptiometry (DEXA) imaging, and computed tomography (CT) scans, as well as additional imaging technology such as radionuclide bone scans for PDB [6]. Specifically, panoramic radiographs [24,25], CT scans of the chest and abdomen [26], simple hip radiographs [27], and even hand radiographs [28] are used to detect osteoporotic changes. Artificial intelligence has emerged as a transformative tool for these bone imaging modalities, enabling automated feature extraction and classification [29]. AI algorithms improve diagnostic accuracy by identifying critical latent variables and rank features that are important for disease characterization [30,31,32,33,34,35,36]. However, AI applications have been primarily used in osteoporosis detection. Few applications have been introduced for detecting PDB changes.

This study aims to fill a critical need by developing an AI-assisted analysis approach for detecting skeletal changes for PDB, and to use this approach to perform a comparative analysis of changes in bones from PDB and osteoporosis mouse models. Specifically, we utilized micro-CT (µCT)-based imaging for rodents combined with AI approaches to characterize bones from a novel OPTN mouse models of PDB that we have developed [19,37], and compare resultant findings to established mouse models of osteoporosis (e.g., ovariectomized and aging models). Using random forest algorithms, we aim to classify disease states and rank cortical and trabecular features by their diagnostic relevance. Additionally, three-dimensional convolutional neural network (3D-CNN) was trained to recognize disease-specific patterns from raw imaging data without the need for manual segmentation by an investigator [38]. Moreover, the advantage of using this AI-assisted approach is the automation of structural analysis of hundreds of bones within a single mouse body. The number of bones and images for analysis will easily be in the thousands when studying cohorts of 5–10 mice of different age, genetic/epigenetic background, and environmental exposures/treatments. Our AI approach overcomes an analysis limit of scalability, automation, and efficiency for high volume of images. Our AI comparative analysis technology may help advance computational methods for skeletal disease classification and screen potential drug compounds in animal models. Our AI approach can be further developed for translational applications to assist radiologists in analyzing high clinical volumes of bone imaging data to detect bone loss, monitor disease progression, and assess response to therapy.

## 2. Materials and Methods

### 2.1. Animals

All animal procedures were approved by the Institutional Animal Care and Use Committee at Ohio State University. Three mouse groups were used—wildtype control (aged mice), Paget’s disease of bone (PDB), and osteoporosis. For the PDB model, global OPTN knockout (KO) mice, consisting of 7 females and 9 males aged 20–26 months on a C57BL/6 background, were generated by crossing Optnflox/flox mice with CMV-Cre mice (Jackson Laboratories, Bar Harbor, ME) as described in our previous study [19,37]. For the postmenopausal osteoporosis model, fifteen C57BL/6 female mice, aged 10 weeks, underwent bilateral ovariectomy (OVX) surgery at Jackson Laboratories (Jackson Laboratories, Bar Harbor, ME), and were euthanized 8 weeks post-surgery at 18 weeks old. For the aging osteoporosis (or control wildtype) model, littermate Optn+/+ wild-type mice were used, including 12 females and 13 males aged 20–26 months [39,40]. The mice were euthanized, one femur per mouse was randomly selected, fixed in 4% paraformaldehyde overnight, and then stored in 70% ethanol for micro-computed tomography (μCT) scanning.

### 2.2. Micro-Computed Tomography (Micro-CT)

Fixed femurs were scanned in 70% ethanol using a micro-CT scanner (µCT50, Scanco Medical AG, Wangen-Brüttisellen, Switzerland) with parameters set at 70 Kvp, 6 µm voxel dimension, 0.5 mm Al filter, and 1200 ms integration time. A standard hydroxyapatite (HA) phantom with density values of 0, 100, 200, 400, and 800 mgHA/cm^3^ was scanned under identical conditions to construct a standard curve for converting grayscale values to absolute bone mineral density (BMD) units. Reconstructed DICOM files were exported for subsequent analysis.

#### Segmentation

**Figure 1:**
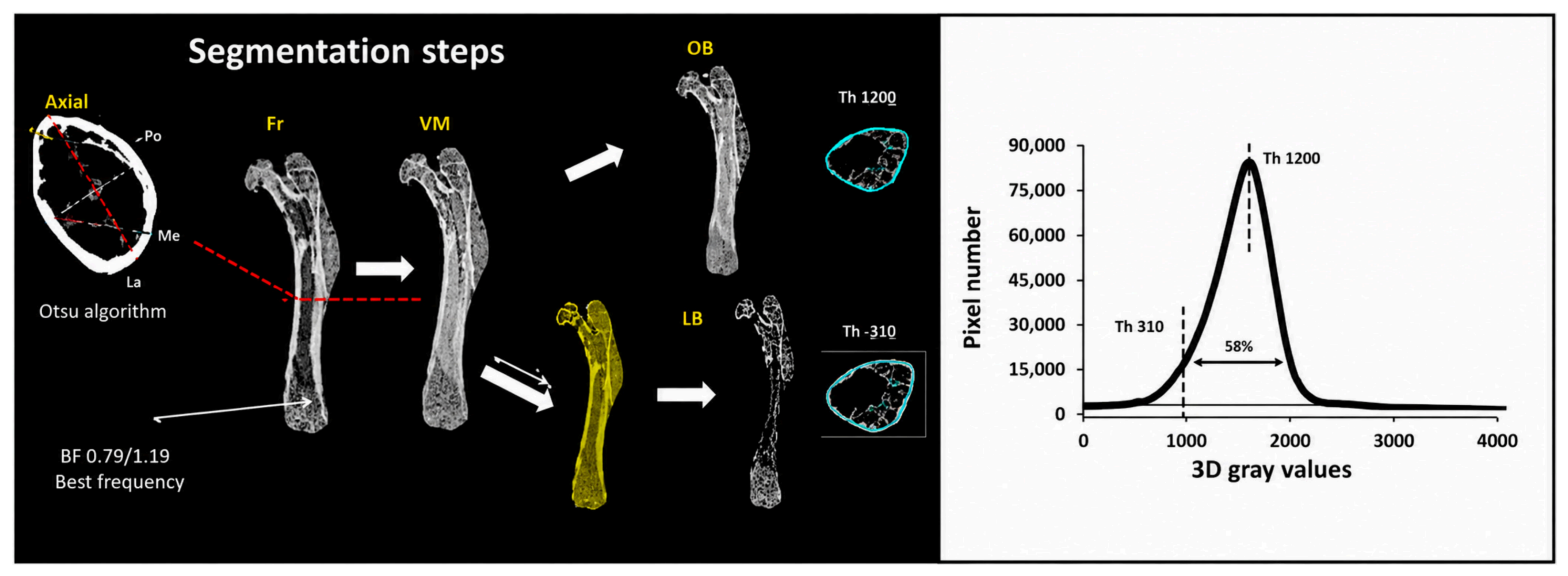
Steps of compartmentation for the 3D microCT image of a femoral bone. CB55 (55% of length) was digitally isolated to calculate CB morphology. A trabecular bone (TB) region (0.72 × 0.72 × 1 mm^3^) above the growth plate at the distal femoral condyle was digitally isolated to compute TB morphology. TMD histograms were used to calculate the mean, lower and upper 5th percentile values.

Bone voxels were segmented from non-bone voxels using a heuristic algorithm described previously [41]. For whole-bone (WB) analysis, the bone volume (BV) was determined by counting all voxels corresponding to the whole bone (WB). Bone mineral density (BMD) was calculated by dividing the total sum of tissue mineral density (TMD) within each bone voxel by the total volume (TV), which included bone, pores, and marrow cavities. Cortical bone (CB) was digitally separated from trabecular bone (TB) by subtracting masked regions from the entire femur (Figure 1). For each region of WB, CB and TB, TMD histograms were used to calculate the mean, lower, and upper 5th percentile values (Low5 and High5, respectively) (Figure 1).

For cortical bone (CB) analysis, a cross-sectioned cortical region at 55% (CB55) of the femoral length from the head was digitally isolated with a thickness of 50 voxels (0.5 mm) (Figure 1). The parameters assessed for CB55 included TMD distribution, cortical bone volume (BV55), total volume (TV55), fraction (BV55/TV55), cortical thickness (Ct.Th), periosteal perimeter (Periosteal Perimeter55), endosteal perimeter (Endosteal Perimeter55), outer and inner diameters of anterior–posterior axis and medial–lateral axis (DAP_outer, DAP_inner, DML_outer, and DML_inner).

For trabecular bone (TB), a region measuring 0.72 × 0.72 × 1 mm^3^, located above the growth plate at the distal femoral condyle, was digitally isolated to analyze TB morphology. Trabecular morphologies including trabecular bone volume (BV), bone surface area (BS), surface-to-volume ratio (BS/BV), number (Tb.N), thickness (Tb.Th), separation (Tb.Sp), connectivity density (Conn.Dn), degree of anisotropy (DA) and structure model index (SMI) were computed.

### 2.3. AI Approaches

#### 2.3.1. Machine Learning (ML)—Random Forest Classifier

Random forest is a supervised AI algorithm that constructs and combines multiple decision trees to form a “forest” [42]. Each tree begins at a single root and branches into two pathways, with each branch representing a distinct outcome. The final classification or output is determined by aggregating the most common prediction across the terminal branches of all the trees [43,44]. In this study, random forest (RF) was selected due to its strong performance with tabular data and its interpretability in various AI applications [45].

A RF algorithm was trained to classify PDB (KO) versus osteoporosis (Aging or OVX) using 10 cortical bone features and 12 trabecular bone features, as listed in Table 1. The model utilized the “leave-one-out” cross-validation approach, where each data point was iteratively used as the test set while the remaining data served as the training set. For comparison, logistic regression (LR) and linear discriminant analysis (LDA) classifiers were trained on the identical feature sets using the same leave-one-out cross-validation scheme. For these linear models, features were standardized using statistics computed within each training fold only. The model’s performance was evaluated using metrics including accuracy (ACC), sensitivity (SEN), specificity (SPE), positive predictive value (PPV), and negative predictive value (NPV). Sensitivity was defined as the proportion of KO bones correctly classified by the model; whereas, specificity was defined as the proportion of Aging or OVX bones correctly classified. Positive predictive value (PPV) represented the proportion of true KO bones among all samples predicted as KO, and negative predictive value (NPV) represented the proportion of true Aging or OVX bones among all samples predicted as Aging or OVX. Additionally, feature importance ranking was performed using the RF algorithm to identify the most critical features contributing to bone disease classification.

#### 2.3.2. Deep Learning (DL)—3D Convolutional Neural Network (3D-CNN) Classifier

**Figure 2:**
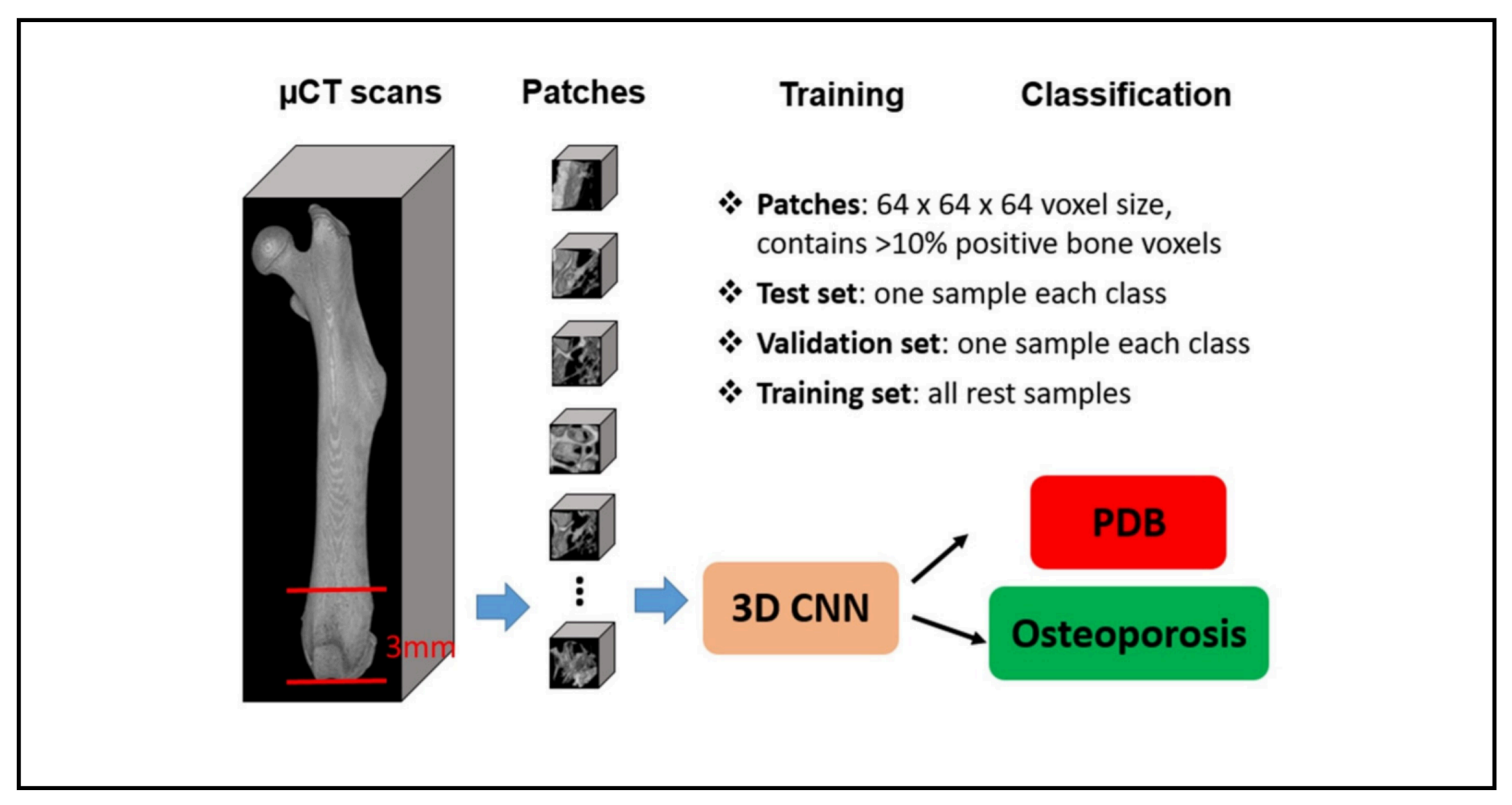
Schematic of the segmentation, training, validation and testing process. The distal 3 mm femoral segment was extracted from µCT images and divided into 64 × 64 × 64 voxel patches. Patches containing ≥10% bone voxels were selected for analysis. The 3D-CNN model was trained, validated, and tested using leave-one-femur-out cross-validation, with femur-level partitioning performed before patch extraction, until all femurs had been evaluated.

A 3 mm femoral segment from the distal end was extracted from µCT images and divided into patches of 64 × 64 × 64 voxels, as illustrated in Figure 2. Patches containing at least 10% positive bone voxels were deemed valid and included in the subsequent analysis. The 3D convolutional neural network (3D-CNN) was trained using a supervised learning approach with leave-one-femur-out cross-validation (LOOCV). Data were partitioned at the femur level before patch extraction: every patch inherited the partition assignment of its parent femur, so that no femur contributed patches to more than one partition within a fold. In each fold, a single femur was held out as the test set. Among the remaining femurs, approximately 20% of the samples from each class were randomly assigned to the validation set, and all remaining femurs were used for training. Network weights were re-initialized at the start of every fold, and the checkout with the lowest validation loss was selected for evaluation on the held-out test femur. The procedure was repeated until every femur had served once as the test set, giving 41 folds for the KO vs. Aging comparison (16 + 25 femurs) and 31 folds for the KO vs. OVX comparison (16 + 15 femurs).

The image branch received one normalized single-channel 64 × 64 × 64 µCT patch. It contained four 3D convolutional blocks with output channels 64, 64, 128, and 256. Each convolution used a 3 × 3 × 3 kernel, stride 1, and padding 1, followed by ReLU, 2 × 2 × 2 max pooling, and 3D batch normalization. Dropout (*p* = 0.30) followed the third block, and global mean pooling produced a 256-dimensional image vector. The implementation retained a 22-feature auxiliary branch (22 -> 32 -> 64 -> 64), but all auxiliary inputs were set to zero for the image-only results. The concatenated representation was classified by two fully connected layers (320 -> 32 -> 2), with ReLU activation and dropout 0.30 between them. The network contained 1,237,122 trainable parameters.

In DL, models are typically developed (“trained”) to identify the optimal configuration, which is then selected (“validated”) based on network parameter optimization. The validated model, representing the most efficient network configuration, is subsequently tested using new, unseen data to assess its performance and generalizability [46].

Training used unweighted two-class cross-entropy and Adam (learning rate 0.0001, AMSGrad enabled), batch size 16, and 200 epochs, without image augmentation. Mean classification accuracy and variance across folds were calculated to evaluate the stability of the 3D-CNN model under repeated validation.

The model’s performance was evaluated using the same metrics as the RF, including sensitivity (SEN), specificity (SPE), overall accuracy (ACC), positive predictive value (PPV), and negative predictive value (NPV).

### 2.4. Statistical Analysis

Analysis of covariance (ANCOVA) followed by Tukey’s post hoc test was used to examine the interactions among experimental groups (OPTN KO, Aging, and OVX) across all bone quality parameters. A significance level of 95% was used (*p* < 0.05). For RF and 3D-CNN, the performance of all schemes was evaluated using these metrics: sensitivity (SEN), specificity (SPE), positive predictive value (PPV), negative predictive value (NPV) and accuracy (ACC).

## 3. Results

### 3.1. Micro-CT Image Manual Segmentation

**Figure 3:**
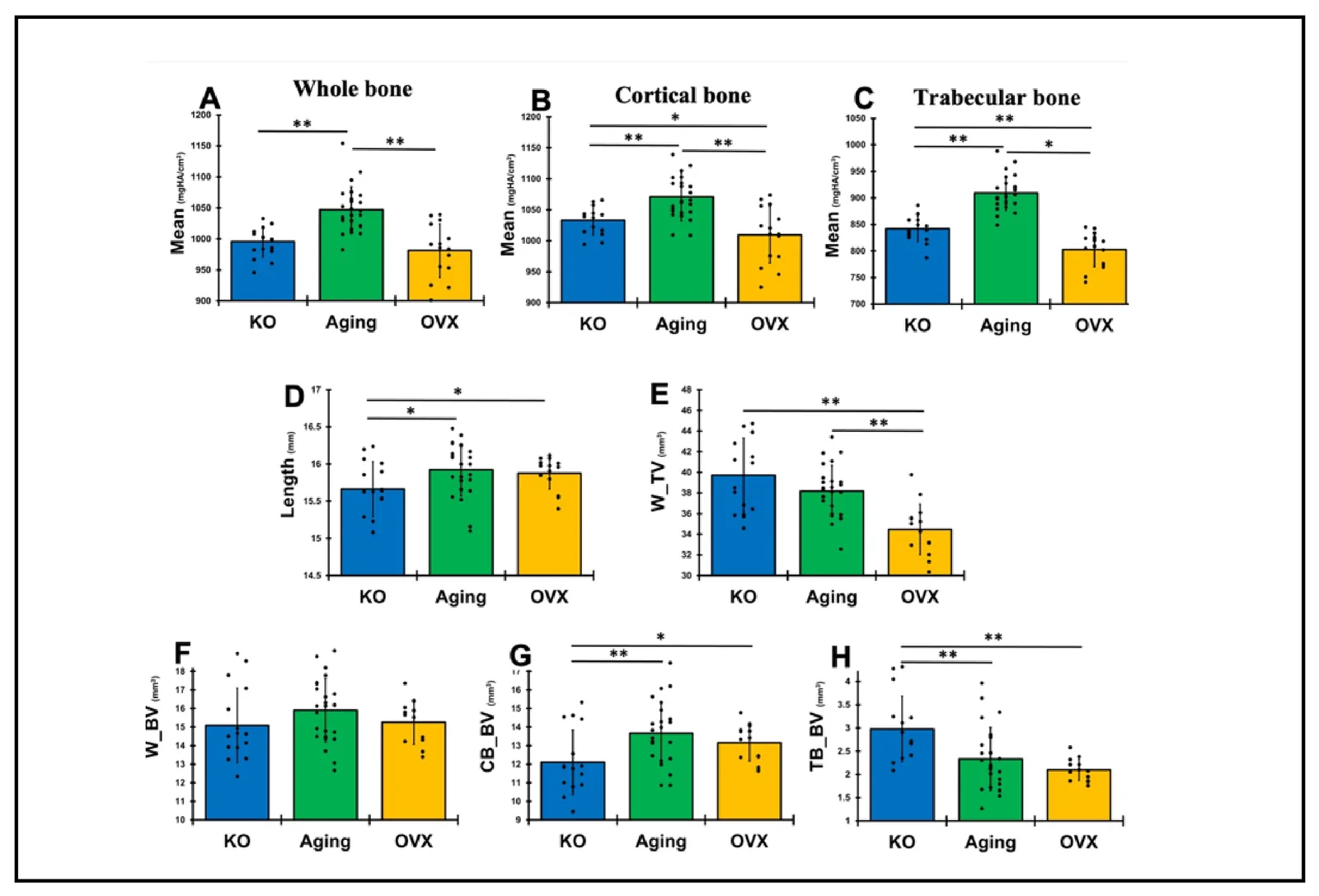
Bone density parameters of (**A**) whole-bone, (**B**) cortical bone and (**C**) trabecular bone and bone volumetric parameters of (**D**) bone length, (**E**) total volume (TV), (**F**) whole bone volume, (**G**) cortical bone volume and (**H**) trabecular bone volume for all experimental groups (* *p* < 0.05 and ** *p* < 0.01), KO, *n* = 16; Aging, *n* = 25, OVX, *n* = 15.

We first performed manual segmentation of micro-CT images to provide training data for AI analysis. Bone density measurements for whole bone, cortical bone, and trabecular bone (Figure 3A–C) were first analyzed. The KO group exhibited significantly lower whole-bone density compared to the Aging group (*p* < 0.05); however, no significant difference was observed when compared to the OVX group (Figure 3A). For cortical and trabecular bone density, the KO group showed lower values than the Aging but higher than OVX (Figure 3B,C).

Next, bone volumetric measurements, including femur length, total volume (TV), and bone volume (BV) (Figure 3D–H) were performed. Bone volumetric analysis showed that the KO femurs were significantly shorter than those of the Aging and OVX groups (Figure 3D). However, the whole-bone total volume (W_TV), which includes both BV and the empty bone marrow space, was significantly greater in the KO group (Figure 3E), indicating that KO femurs were shorter in length but larger in overall size based on volume. No significant difference was observed in whole-bone BV (W_BV) among the three groups (Figure 3F). However, KO mice had a significantly reduced cortical bone BV (CB_BV) compared to Aging and OVX groups (Figure 3G), and increased trabecular bone BV (TB_BV) (Figure 3H).

**Figure 4:**
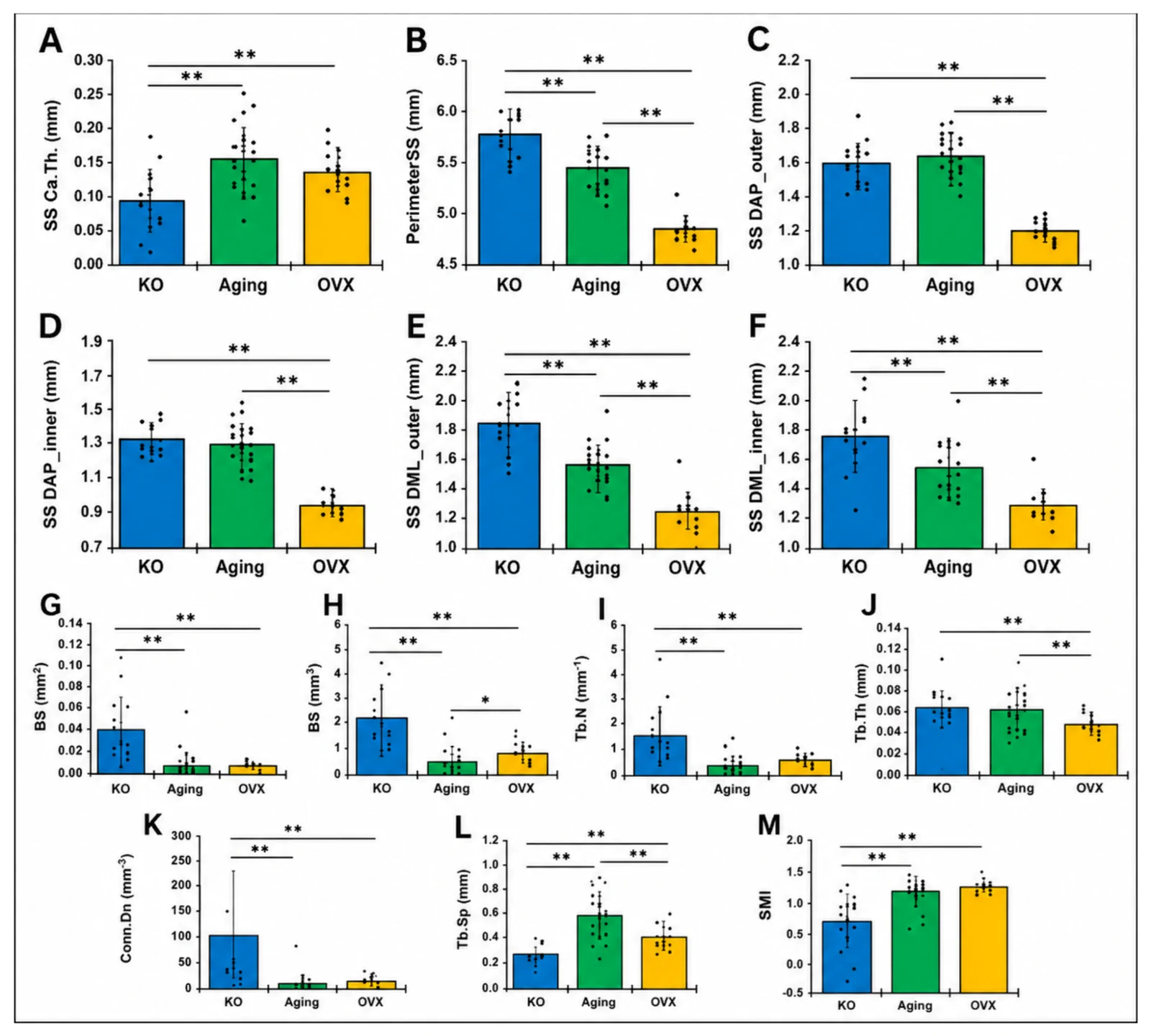
Cortical bone morphological parameters of (**A**) thickness (55 Ct.Th), (**B**) perimeter (Perimeter55), (**C**) diameter in AP_outer (55 DAP_outer), (**D**) diameter in AP_inner (55 DAP_inner), (**E**) diameter in ML_outer (55 DML_outer), (**F**) diameter in ML_inner (55 DML_inner) and trabecular bone morphological parameters of (**G**) bone volume (BV), (**H**) bone surface area (BS), (**I**) trabecular unit number (Tb. N), (**J**) trabecular unit thickness (Tb. Th), (**K**) connectivity density (Conn.Dn), (**L**) separation (Tb.Sp), and (**M**) structure model index (SMI) from each experimental group (* *p* < 0.05 and ** *p* < 0.01). (KO, *n* = 16; Aging, *n* = 25, OVX, *n* = 15).

Morphological analysis (Figure 4) showed that the KO group had significantly reduced cortical bone thickness (55 Ct.Th, Figure 4A) but significantly increased perimeter (Perimeter55, Figure 4B), as well as outer and inner diameters in DML orientation (55 DML_inner and 55 DML_outer) (Figure 4C–F). No significant differences were observed in DAP_inner and DAP_outer between the KO and Aging groups (Figure 4D,E).

For morphological features of trabecular bone (Figure 4G–M), the KO group exhibited significantly higher bone volume (BV), bone surface area (BS), trabecular unit number (Tb.N) and trabecular unit connectivity density (Conn.Dn) (Figure 4G–I,K), but significantly reduced parameters of trabecular unit separation (Tb.Sp) and structure model index (SMI) (Figure 4L,M), compared to both osteoporosis groups. Trabecular bone thickness (Tb.Th), was significantly greater in KO than OVX but similar to the Aging group (Figure 4J).

### 3.2. Machine Learning—Random Forest Classifier

Data derived from µCT segmentation and analysis was then used to train our AI models and determine how well these AI approaches were able to classify and distinguish bone changes in our mouse models of PDB and osteoporosis. We began by evaluating the performance of the Random Forest (RF) approach. As summarized in Table 2, when comparing “KO vs. Aging” datasets, an accuracy (ACC) of 83% was achieved using CB features, which increased to 90% when TB features were used. In the “KO vs. OVX” dataset, the ACC was 97% based on CB features and 94% based on TB features. Sensitivity (SEN), specificity (SPE), positive predictive value (PPV), and negative predictive value (NPV) for these models are detailed in Table 2. A baseline comparison against logistic regression and linear discriminant analysis, trained on the same features and cross-validation, showed comparable classification performance. Random Forest was chosen primarily for its interpretable feature-importance ranking.

**Figure 5:**
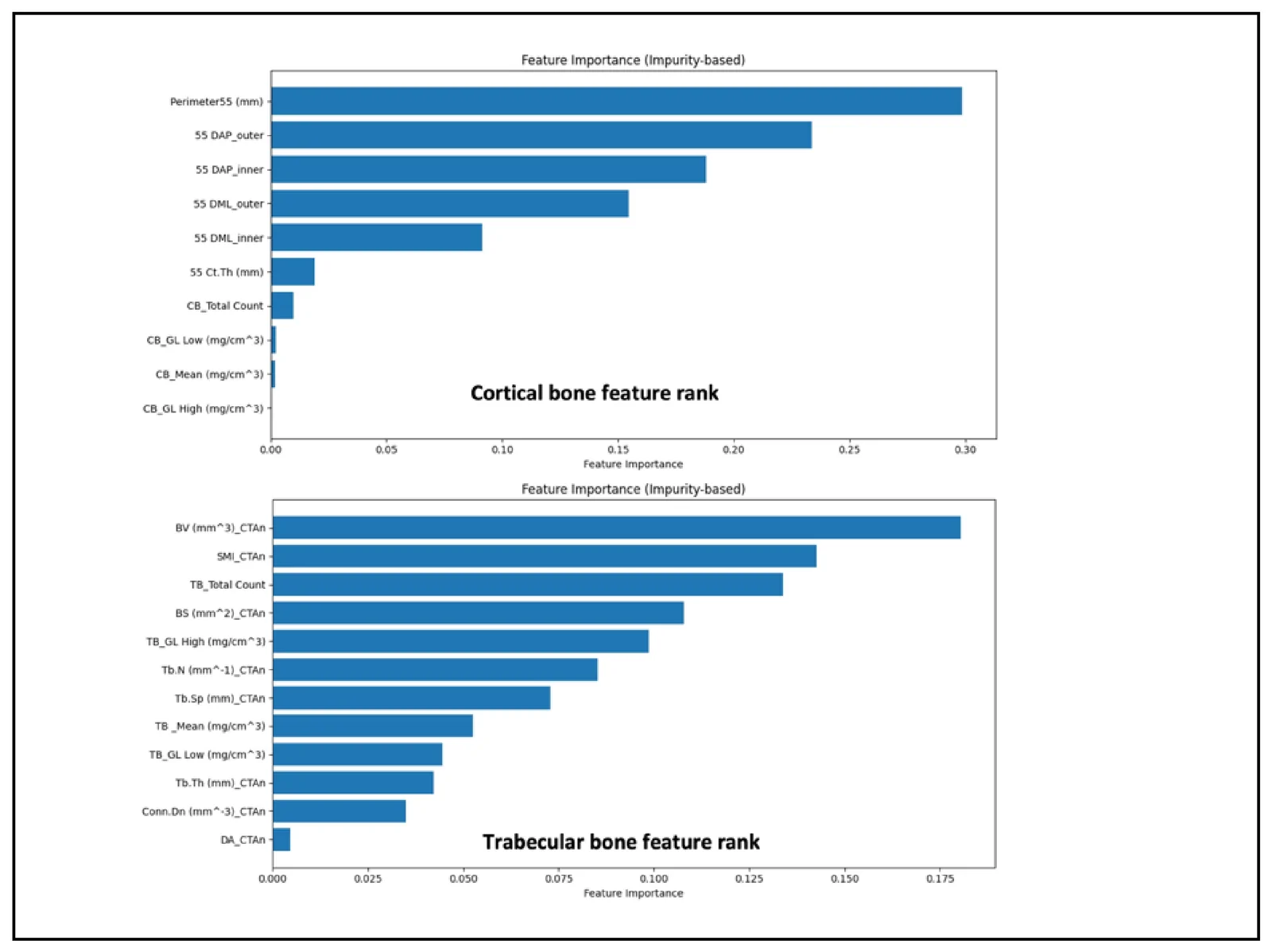
Feature rank calculated by RF for KO vs. Aging. The *x*-axis represents feature importance and the *y*-axis represents input features (variables). Input features receive a score ranging from 0 to 1, and a higher score representing more importance.

**Figure 6:**
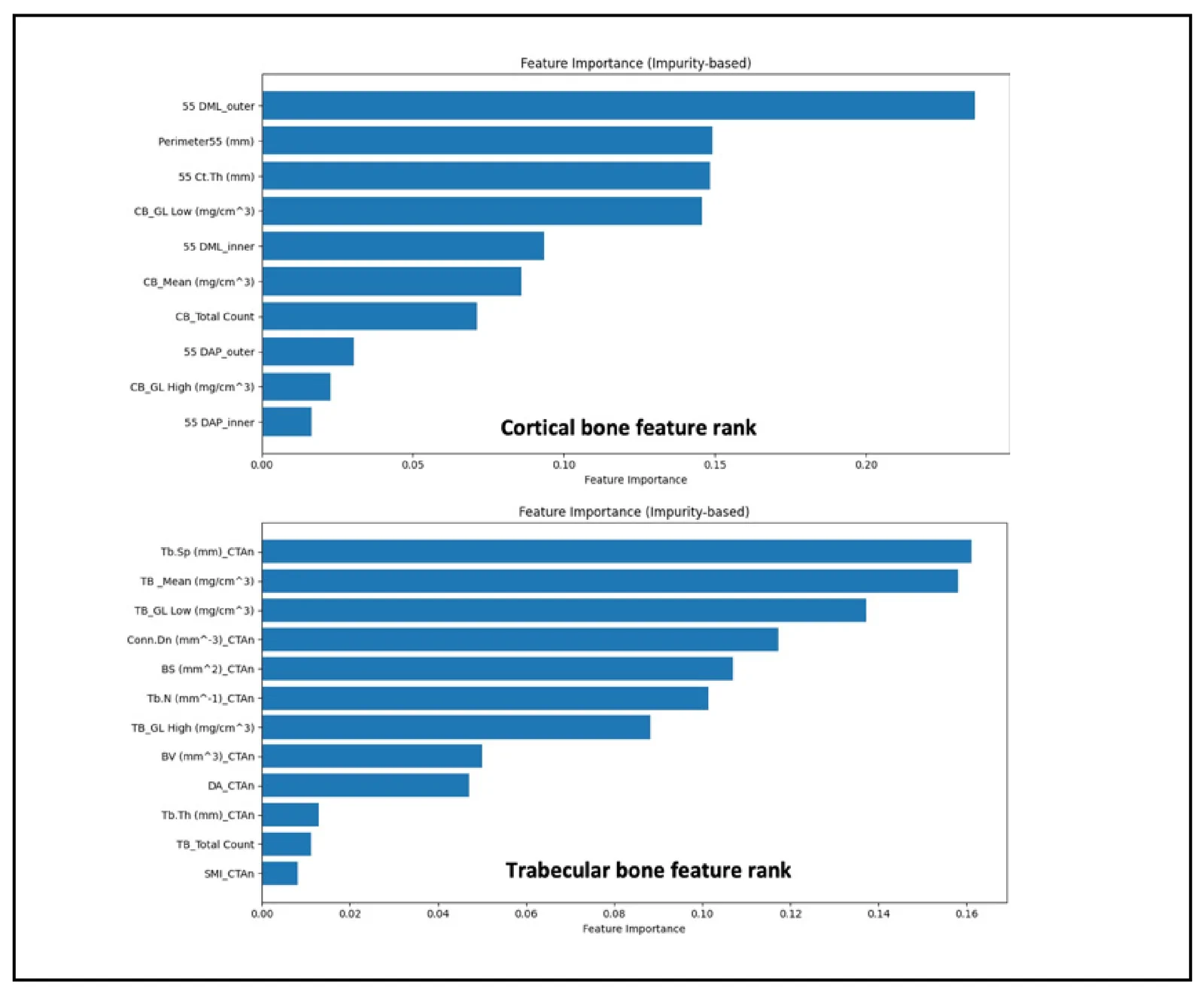
Feature rank calculated by RF for KO vs. OVX. The *x*-axis represents feature importance and the *y*-axis represents input features (variables). Input features receive a score ranging from 0 to 1, and a higher score representing more importance. In both datasets, the CB morphological parameters, particularly the perimeter and the outer/inner diameters in the medial–lateral orientation, consistently ranked as the most important features. Among TB parameters, BS emerged as the top-ranked feature across analyses.

The RF algorithm, in addition to its predictive capabilities, provides insights into the relative importance of input variables for identifying and classifying bone diseases. Feature importance rankings (Figure 5 and Figure 6) highlighted cortical bone perimeter, medial–lateral diameters, and trabecular bone surface area as top contributors.

Figure 5 presents rankings of key features generated by the RF algorithm for the “KO vs. Aging” datasets. For CB features, the most influential variables included morphological parameters of perimeters and the outer and inner diameters in both DAP and DML orientations. Regarding TB features, the highest-ranked variables were BV, SMI, BS, total TB volume (TV), and High5 values.

Figure 6 depicts the feature rankings for the “KO vs. OVX” datasets. For CB features, the algorithm prioritized parameters of the inner and outer diameters in the DML orientation, perimeter, cortical thickness (Ct.Th), and Low5 values. For TB features, the top-ranked variables included trabecular separation (Tb_Sp), mean values, Low5, connectivity density (Conn.Dn), and BS.

### 3.3. Deep Learning—3D Convolutional Neural Network (3D-CNN) Classifier

The RF approach above relies on a person to first manually segment, predefine features, and classify outcomes for AI training. As such, this does not overcome limits of scalability, automation, and efficiency. The AI algorism needs to automatically segment and define key features extracted from bone imaging data. For this purpose, we developed an approach using the 3D convolutional neural network algorithm to enable AI analysis directly from unsegmented raw imaging data.

**Figure 7:**
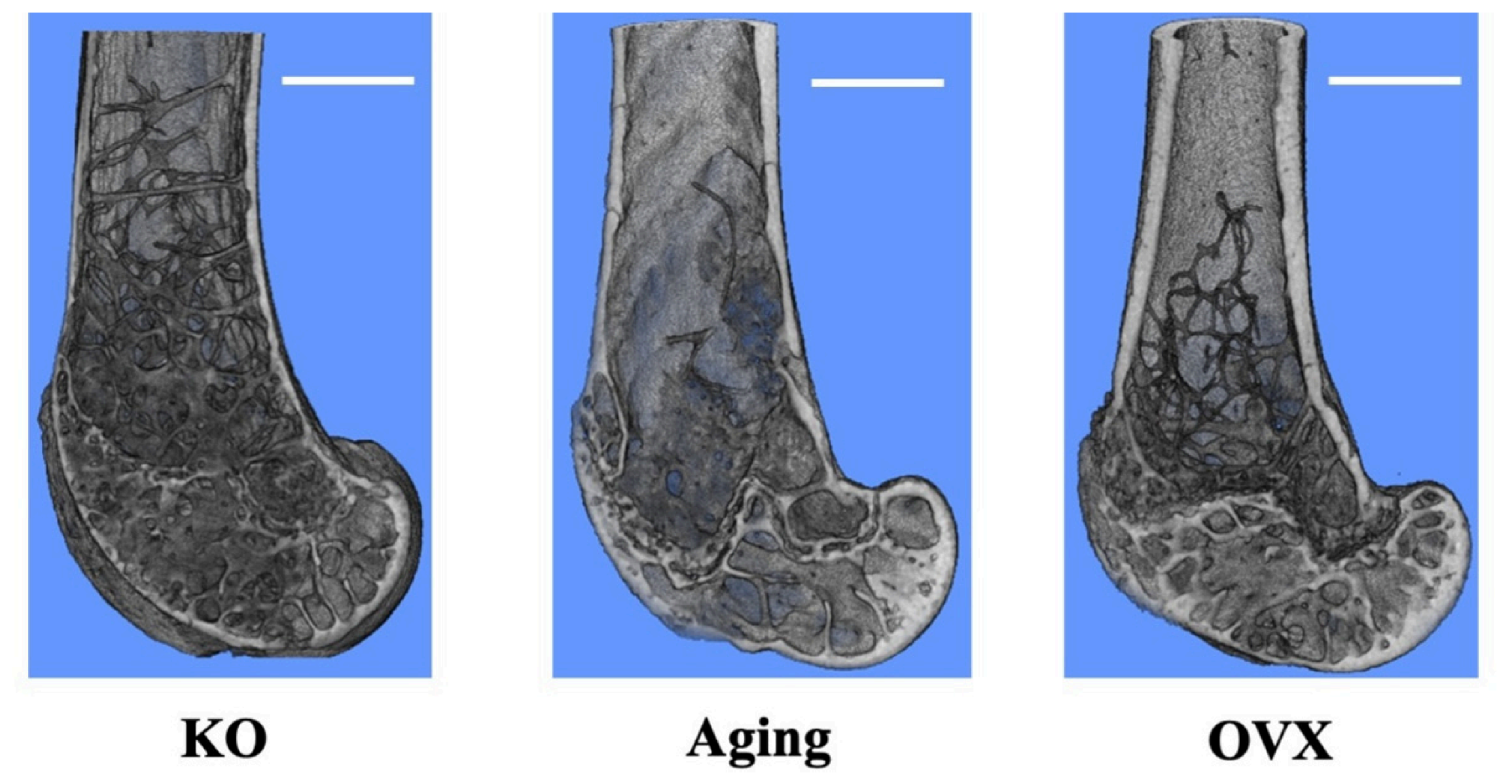
Representative microCT scans of distal femurs each experimental mouse group showing clear phenotypic differences, scale bars represent 1 mm. (KO, *n* = 16; Aging, *n* = 25, OVX, *n* = 15).

Figure 7 illustrates a portion of an image of a femur s from the distal end for each experimental mouse group. Even without AI, one can see significant differences in bone morphology. For trabecular bone, KO demonstrated increased but disorganized trabecular bone at the distal femur, representing an osteosclerotic-like phase of PDB. For cortical bone, KO and Aging groups had reduced cortical bone thickness compared to OVX.

To evaluate the diagnostic performance of the 3D-CNN model, we measured accuracy (ACC) to determine how well the model can correctly identify and classify bone diseases from micro-CT scans. To assure the robustness and generalizability of the 3D-CNN model, leave-one-femur-out cross-validation was applied as described in Section 2.3.2. For the KO versus aging WT comparison, 29 of 41 mice were correctly classified (accuracy, 70.7%; 95% CI, 54.5–83.9%). With KO as the positive class, sensitivity was 50.0% (8/16; 95% CI, 24.7–75.3%), and specificity for aging WT was 84.0% (21/25; 95% CI, 63.9–95.5%) (Table 3).

## 4. Discussion

This study demonstrates the feasibility of machine learning and deep learning approaches, specifically RF and 3D CNN models, in differentiating PDB and osteoporosis using µCT-derived bone images from representative mouse models of these diseases. By integrating traditional morphometric analysis with ML and DL based classification, the study provides novel insights into the phenotypic divergence between these two bone disorders and provides proof-of-concept evidence for AI-based skeletal phenotyping in a preclinical mouse model.

Traditional µCT-based segmentation revealed significant differences in bone architectures, including volumetric, mineral density, and morphological features between PDB and osteoporosis. These features are consistent with literature reports. Specifically, we found that KO mice exhibited significantly higher bone volume but lower mean tissue mineral density in the whole bone, cortical bone and trabecular bone compared with aging mice (Figure 3A–C). This pattern suggests enhanced yet immature bone formation in KO mice, consistent with the pathology of PDB, which involves extensive bone degradation followed by abnormal formation of new immature bone [13]. Regarding volumetric changes (Figure 3D–H), the KO group exhibited significantly reduced femur length but increased total volume compared to both Aging and OVX groups. This finding suggests that KO bones became shorter yet thicker, consistent with clinical features of PDB, [3,14]. Morphological parameters further revealed that KO mice had reduced cortical bone thickness (Figure 4A) but expanded periosteal surfaces from increased cortical bone perimeter, as well as the inner and outer diameters along the medial–lateral and anterior–posterior axes (Figure 4B–F). Trabecular bone analysis revealed that KO mice exhibited significantly increased bone volume, number, and surface area compared to Aging and OVX groups (Figure 4G–I), indicating higher trabecular bone mass. KO mice also showed reduced trabecular separation (Figure 4L) and structure model index (Figure 4M), alongside increased connectivity (Figure 4K) and thickness (Figure 4J), suggesting denser but more irregular and structurally weaker trabecular architecture. Taken together, findings from our phenotypic analysis of KO bones are consistent with disorganized bone formation characteristic of Paget’s disease of bone, as previously reported by Hu et al. [19].

When results of our bone phenotypic analysis were used to train the Random Forest AI classifier, accuracies exceeded 90% in both KO vs. Aging and KO vs. OVX comparisons, with sensitivity of 88– 93% and specificity of 75–100%. Notably, the RF model identified cortical bone perimeter, mediolateral diameters, and trabecular bone surface area as the most critical features for accurate disease classification. The RF classifier, with strong performance for tabular data [45], achieved high accuracy in distinguishing disease types using cortical and trabecular bone features. These findings align with the known structural alterations in PDB and suggest that specific geometric parameters may serve as robust diagnostic markers. To our knowledge, this is the first study to apply a supervised machine learning model to µCT-derived morphometric features for the differentiation of PDB and osteoporotic bone in mouse models.

Despite the RF algorithm’s strong performance, its need to be trained by datasets consisting of manually segmented and predefined features limits its scalability and efficiency. To address this, we explored the application of a deep learning algorithm 3D CNN to classify disease states directly from raw µCT imaging data. The CNN model achieved 68.2% accuracy in classifying KO versus aging bones. These results, though modest, are notable given the limited sample size and restricted region of interest analyzed in this study. They suggest that KO bones are consistently classified by 3D CNN to be distinct from OVX and Aging bones. This finding may have mechanistic implications and support the current hypothesis that PDB phenotypes result from abnormal bone degeneration followed by abnormal bone formation [19]. In contrast, OVX-induced osteoporosis is characterized primarily by bone loss without abnormal bone formation, consistent with findings from a rat study by Liu. et al. [41,47]. Our data showing significant differences between bones from PDB, OVX, and Aging mouse cohorts suggest that these differences detected by our AI approach are attributable to different disease mechanisms for these bone diseases.

Other biological limitations include age differences between KO and OVX, which may affect cortical bone. However, OVX has been used as a standard model to simulate osteoporosis. The comparison of trabecular patterns between KO and OVX should be still valid. Due to the small sample size, the present study does not address gender differences.

The moderate discriminative capability of the 3D CNN model highlights a well-recognized challenge in deep learning applications: the need for large, diverse datasets to optimize performance. Previous studies have demonstrated that CNNs excel in image-based classification tasks when trained on substantial and heterogeneous datasets [48]. Furthermore, the observed variance (0.114) suggests that model performance remains sensitive to data partitioning, reflecting a known limitation of deep learning approaches when applied to relatively small datasets.

Future work should therefore focus on expanding the sample size, incorporating multiple skeletal sites, and augmenting imaging diversity to enhance model generalizability and performance. Our sample size (KO *n* = 16, Aging *n* = 25, OVX *n* = 15) is small, particularly for the 3D-CNN approach. Because this study was exploratory and based on an available animal cohort, an a priori power analysis was not performed before data collection. By using our results, we performed a simulation-based sample size analysis using 10,000 simulated datasets to guide future study design. The simulation indicated that approximately 50 mice per group would achieve an estimated margin of error of approximately 8%, around 90% classification accuracy at the 95% confidence level. Future larger cohorts, including age-matched and sex-balanced samples, will be required to improve statistical power and further validate the proposed AI models.

Importantly, many AI applications do not reveal specific features of the dataset used by the AI to yield classification findings. The dataset is essentially fed into a “black box.” In contrast to this, our work integrated the ability to identify and rank features of the dataset that are important and used by the RF classifier to generate classification results. This method assesses the impact of individual features on the model’s predictions [44]. In both KO vs. Aging and KO vs. OVX comparisons, cortical bone perimeter and mediolateral diameters emerged as the top predictive features, along with trabecular bone surface area. These parameters align with the pathological hallmarks of PDB and highlight AI’s capacity to prioritize biologically relevant features, which is critical for bridging computational outputs with mechanistic understanding in translational bone research.

## 5. Conclusions

In conclusion, this study establishes a foundational framework for applying AI to characterize PDB and osteoporosis bone morphology and structure. The RF algorithm demonstrated high accuracy and interpretability using µCT-derived morphometric features that were first manually segmented. To remove the need for manual data processing, we applied the 3D CNN model, which provided proof-of-concept for using raw imaging data directly. These findings highlight AI’s potential to enhance diagnostic efficiency, improve understanding of skeletal disease mechanisms, and development of novel therapeutics for these disabling diseases.

## Figures and Tables

**Table 1:** Feature definitions.

Features		Description
Volumetric	CB_total count	Cortical bone total voxel count
	TB_total count	Trabecular bone total voxel count
Mineral Density	CB_Mean (mg/mm^3^)	Cortical bone mean values of tissue mineral density (TMD)
	CB_Low_5_ (mg/mm^3^)	Cortical bone lower 5th percentile values of TMD histogram
	CB_High_5_ (mg/mm^3^)	Cortical bone higher 5th percentile values of TMD histogram
	TB_Mean (mg/mm^3^)	Trabecular bone mean values of tissue mineral density (TMD)
	TB_Low_5_ (mg/mm^3^)	Trabecular bone lower 5th percentile values of TMD histogram
	TB_High_5_ (mg/mm^3^)	Trabecular bone higher 5th percentile values of TMD histogram
Morphology	Ct.Th (mm)	Cortical bone thickness at 55% of the femoral length from the head
	Perimeter (mm)	Cortical bone perimeter at 55% of the femoral length from the head
	DAP (mm)	Diameters of anterior–posterior axis at 55% of the femoral length from the head
	DML (mm)	Diameters of medial–lateral axis at 55% of the femoral length from the head
	BV (mm^3^)	Trabecular bone volume
	BS (mm^2^)	Trabecular bone surface
	BS/BV (mm^−1^)	Trabecular surface-to-volume ratio
	Tb.N (mm^−1^)	Trabecular number
	Tb.Th (mm)	Trabecular thickness
	Tb.Sp (mm)	Trabecular separation
	Conn.Dn (mm^−3^)	Trabecular connectivity density
	SMI	Structure model index
	DA	Degree of anisotropy

**Table 2:** Comparison of performances.

	Accuracy (ACC)	Sensitivity (SEN)	Specificity (SPE)	PPV	NPV
KO vs. Aging (CB features)	0.83	0.75	0.88	0.800	0.85
KO vs. Aging (TB features)	0.90	0.94	0.88	0.83	0.96
KO vs. OVX (CB features)	0.97	1	0.93	0.94	1
KO vs. OVX (TB features)	0.94	0.94	0.93	0.94	0.93

**Table 3:** Comparison of performances by mouse majority vote.

Comparison and Group	ACC (95% CI)	SEN for Aging (95% CI)	SPE for KO (95% CI)	Independent Test Group
KO vs. Aging	70.7% (54.5–83.9%)	50.0% (24.7–75.3%)	84.0% (63.9–95.5%)	16 KO+ 25 aging
KO vs. OVX	77.4% (58.9–90.4%)	81.3% (54.4–96.0%)	73.3% (44.9–92.2)	16 KO+ 15 OVX

Note. Values are mouse-level estimates from leave-one-femur-out cross-validation (LOOCV). Ninety-five percent confidence intervals are two-sided Clopper–Pearson exact binomial intervals. Sensitivity is recall for KO; specificity is recall for aging WT and OVX. Patch outputs are descriptive supplementary results because patches from one mouse are correlated. Intervals are conditional on the fixed cross-validation procedure and are not external-validation intervals. ACC—accuracy; SEN—sensitivity; SPE—specificity; CI—confidence interval.

## Data Availability

The data set and AI programs presented in this study are available from the corresponding author upon reasonable request.
